# Volatile metabolites from new cultivars of catnip and oregano as potential antibacterial and insect repellent agents

**DOI:** 10.3389/fpls.2023.1124305

**Published:** 2023-02-23

**Authors:** Harna K. Patel, Erik Nunes Gomes, Qingli Wu, Nrupali Patel, Donald Y. Kobayashi, Changlu Wang, James E. Simon

**Affiliations:** ^1^ New Use Agriculture and Natural Plant Products Program, Department of Plant Biology, Rutgers University, New Brunswick, NJ, United States; ^2^ Department of Plant Biology, Rutgers University, New Brunswick, NJ, United States; ^3^ Federal Agency for Support and Evaluation of Graduate Education (CAPES), Ministry of Education of Brazil, Brasilia, DF, Brazil; ^4^ Department of Medicinal Chemistry, Ernest Mario School of Pharmacy, Piscataway, NJ, United States; ^5^ Department of Entomology, Rutgers University, New Brunswick, NJ, United States; ^6^ Center for Agricultural Food Ecosystems, Institute of Food, Nutrition & Health, Rutgers University, New Brunswick, NJ, United States

**Keywords:** carvacrol, essential oil, nepetalactone, urban pest, bacterial plant pathogen, *Pseudomonas*, *Xanthomonas*, *Cimex lectularius*

## Abstract

Plant based natural products have been widely used as antibacterial and insect repellent agents globally. Because of growing resistance in bacterial plant pathogens and urban pests to current methods of control, combined with the long- and short-term negative impact of certain chemical controls in humans, non-target organisms, and the environment, finding alternative methods is necessary to prevent and/or mitigate losses caused by these pathogens and pests. The antibacterial and insect repellent activities of essential oils of novel cultivars of catnip (*Nepeta cataria* L. cv. CR9) and oregano (*Origanum vulgare* L. cv. Pierre) rich in the terpenes nepetalactone and carvacrol, respectively, were evaluated using the agar well diffusion assay and petri dish repellency assay. The essential oils exhibit moderate to high antibacterial activity against three plant pathogens, *Pseudomonas cichorii*, *Pseudomonas syringae* and *Xanthomonas perforans* of economic interest and the individual essential oils, their mixtures and carvacrol possess strong insect repellent activity against the common bed bug (*Cimex lectularius* L.), an urban pest of major significance to public health. In this study, the essential oils of catnip and oregano were determined to be promising candidates for further evaluation and development as antibacterial agents and plant-based insect repellents with applications in agriculture and urban pest management.

## Introduction

Rapid population growth and increased urbanization are characteristics of modern society, making it imperative to address issues such as quality of life and food security (Ruel et al., 2017; Mouratidis, 2021). In this context, the nature of ecological interactions in agricultural and urban settings needs to be adapted to meet the demands of globalized society (Li et al., 2022). Plant natural products have a remarkable potential to develop sustainable food production systems as well as support human well-being. These products have been long used as antimicrobial agents and insect repellents (Mahady et al., 2008; da Silva and Ricci-Júnior, 2020). Among the many natural products, there has been an increased interest in using essential oils from plants as alternative options for pest management in both developed and developing countries, with the worldwide bioinsecticide market projected to grow from $2.2 billion in 2020 to $4.6 billion by 2025 (Mossa, 2016; Devrnja et al., 2022). Essential oils are volatile plant secondary metabolites that are composed of a complex blend of various bioactive compounds such as monoterpenes, sesquiterpenes and their oxygenated derivatives (Dhifi et al., 2016). Lamiaceae (mint family) species, frequently used in the food, cosmetics, and pharmaceutical industries, are some of the most economically important aromatic plants, producing a variety of volatile compounds that have been known to possess attractant, repellent, antifeedant, larvicidal, insecticidal, antimicrobial, and antioxidant activities among others (Nieto, 2017; Giatropoulos et al., 2018; Ebadollahi et al., 2020; Ramos da Silva et al., 2021; Nazar et al., 2022). For management of insect pests, plants from the *Origanum, Thymus, Satureja, Lavandula, Rosmarinus, Melissa, Ocimum, Salvia* among many other genera in the Lamiaceae family have been used because of their bioactive aromatic volatiles that exhibit activity against a variety of pests (Ebadollahi et al., 2020).

Two well-recognized plants from the Lamiaceae family, oregano (*O. vulgare*) and catnip (*N. cataria*) and, have been reported to exhibit a wide range of bioactivities, including antibacterial and insect repellent activities (Suschke et al., 2006; Peterson and Coats, 2011; Leyva-López et al., 2017; Sharififard et al., 2018; Gomes et al., 2021). Oregano essential oil has shown antibacterial activity against foodborne and other pathogenic bacteria such as *Escherichia coli, Bacillus cereus*, *Salmonella enteritidis, Listeria monocytogenes, Vibrio vulnificus, Clostridium perfringens*, and *Staphylococcus aureus*, leading to opportunities for its use as an antibacterial agent in the food and poultry industries (Kosakowska et al., 2021; Jin et al., 2022; Luo et al., 2022). In addition, use of oregano essential oil for managing pests of importance has also been studied. Oregano essential oil has been reported to have either lethal or sublethal activity against major pests of importance such as the maize weevil (*Sitophilus zeamais*) (Eesiah et al., 2022), stored product weevil (*Sitophilus granarius*) (Plata-Rueda et al., 2022), red flour beetle (*Tribolium castaneum*) (Licciardello et al., 2013), blowfly (*Calliphora vomitoria*) (Bedini et al., 2021), bed bugs (*Cimex lectularius*) (Sharififard et al., 2018), mosquitoes (*Aedes albopictus*) (Giatropoulos et al., 2022) and ticks (*Ixodes ricinus*) (Soutar et al., 2019).

Similarly, catnip essential oil has shown antibacterial activity against pathogens and pests of importance to human and animal health such as *E. coli, S. aureus, Pseudomonas aeruginosa, Enterococcus faecalis, Citrobacter freundii*, *Branhamella ovis, Serratia marcescens* and *Neisseria subflava* among others (Ghosh et al., 2021) and insect repellent activity against important pests such as stable flies (*Stomoxys calcitrans*) and houseflies (*Musa domestica*) as well as American cockroaches (*Periplaneta americana*), red poultry mites (*Dermanyssus gallinae*), and disease vectoring mosquitoes (*Anopheles gambiae*, *Aedes aegypti* and *Culex quinquefasciatus*) (Schultz et al., 2004; Zhu et al., 2009; Birkett et al., 2011; Zhu et al., 2014; Reichert et al., 2019). The antibacterial and insect repellent activities of oregano and catnip can be attributed to the major volatile terpene compounds such as carvacrol and nepetalactone respectively, found in the essential oil of the plants (Lambert et al., 2001; Peterson and Coats, 2011; Evergetis et al., 2018; Ghosh et al., 2021). Oregano and catnip essential oils have been registered with the Environmental Protection Agency (EPA) in the United States for use as a biochemical pesticide and as an insect repellent respectively (Environmental Protection Agency, 2010; Environmental Protection Agency, 2011). These two plants can be of strategic importance in mitigating damages related to plant pathogens and insect pests.

*Xanthomonas* and *Pseudomonas* are two genera of plant pathogenic bacteria that pose a serious threat to food security (Mansfield et al., 2012). *X. perforans* is known to cause bacterial spot, one of the most destructive diseases affecting economically valuable crops in the Solanaceae family, including tomatoes and peppers (Srivastava et al., 2021). *P. syringae* is one of the most common plant pathogens containing over 60 pathovars with distinct host plants, which collectively infect almost all commercially valuable crops (Xin et al., 2018). *P. cichorii* has a wide host range similar to *P. syringae* and it has been reported to cause bacterial spot on crops such as lettuce, escarole, and sweet basil plants in New Jersey (Xin et al., 2018; Patel et al., 2019; Patel et al., 2021). Bactericides, especially antibiotics and copper-based formulations, are widely used for controlling bacterial diseases of plants (Miller et al., 2022). However, efficacies of these controls have diminished substantially due to the ease at which resistance develops within bacterial pathogen populations (Sundin and Wang, 2018; Fan et al., 2022). Additional concerns regarding use of copper-based formulations include unfavorable effects on the environment such as decreased soil fertility, horizontal transfer of resistance genes to non-target microorganisms and phytotoxicity in plants, leading to a reduction in the quality and yield of the crop (Sundin et al., 2016; Lamichhane et al., 2018), highlighting the serious need for alternative methods of controlling bacterial plant diseases.

Similar to plant pathogens, arthropods such as common bed bugs (*Cimex* spp.), a well-known urban pest, are also of significant importance to food production and human well-being (Axtell, 1999; Zorrilla-Vaca et al., 2015). In addition to directly affecting humans, the common bed bug also poses a threat to the food industry, specifically the poultry industry (Kulash, 1947; Axtell, 1999). As a result of bed bug infestations in poultry farms, egg production yields have been reported to be decreased by up to 10% (Cater et al., 2011). Also, bed bugs can be transferred from infested poultry farms to domestic locations by poultry workers (Steelman et al., 2008). In addition to facing the significant cost of bed bug treatment and removal, those affected can also experience adverse effects on their mental and physical health as well as their overall quality of life (Scarpino and Althouse, 2019). Furthermore, increasing resistance has been reported in bed bug populations to various insecticides, including pyrethroids (Davies et al., 2012). Bed bugs in poultry breeding operations are incredibly difficult to control, especially in the presence of poultry due to limited availability of products safe for use in the presence of poultry (Tabler et al., 2015). Once established in a suitable environment, the common bed bug is challenging to control and nearly impossible to eliminate from poultry houses (Kulash, 1947; Tabler et al., 2015). Thus, additional strategies for control as well as more sustainable methods to overcome resistance in bed bugs are needed.

One of the challenges associated with the development of sustainable essential oil-based products is a steady supply of raw materials. Therefore, availability of genetic materials for specialty crops that produce high amounts of bioactive compounds is a vital aspect that needs to be addressed in the supply chain. As part of the effort to develop high essential oil yielding genetic materials, breeding programs have been focused on the Lamiaceae family to produce improved varieties with desirable phytochemical profiles that are adapted to different growing conditions. Among these new plants, a new patented cultivar of *N. cataria, CR9*, and a new patented cultivar of *O. vulgare*, Pierre, have been developed to produce high yield of essential oil and content of (*Z,E*)-nepetalactone and carvacrol respectively (Reichert et al., 2016; Reichert et al., 2021). These cultivars are some of the first for these species to be adapted to the North American and Mid-Atlantic regions and possess an upright growth habit allowing for a mechanical harvest and large-scale production (Reichert et al., 2016; Reichert et al., 2021).

While catnip and oregano essential oils have previously been tested against pathogens and pests of importance to agriculture and human health (Vasinauskiene et al., 2006; Peterson and Coats, 2011; Zomorodian et al., 2012; La Pergola et al., 2017), the study of antimicrobial and insect repellent effects of novel catnip cv. CR9 and oregano cv. Pierre remain underexplored. In addition, carvacrol-rich oregano essential oil has not been tested against *P. cichorii* and *C. lectularius* and catnip essential oil has not been tested against *P. cichorii* and *X. perforans*. Therefore, the aim of this study was to evaluate the chemical composition and potential of the essential oils of the new and unique *N. cataria* cv. CR9 and *O. vulgare* cv. Pierre as antibacterial agents against *P. cichorii*, *P. syringae*, and *X. perforans*; essential oil of *O. vulgare* cv. Pierre and mixtures of the essential oil of *O. vulgare* cv. Pierre and *N. cataria* cv. CR9 as insect repellents against *C. lectularius*.

## Material and methods

### Plant materials, essential oil extraction and sample preparation

Seedlings of catnip cv. CR9 were grown under greenhouse conditions in the spring and transplanted in the field in late May of 2020 once they reached 15-20 cm in height at the Clifford E. and Melda C. Snyder Research Farm in Pittstown, NJ. The growing conditions after transplant are the same as described by (Patel et al., 2022). The plants were harvested in the full flowering stage at the end of July and dried at 37°C in a forced air walk-in dryer onsite at the farm. Vegetative clones of the oregano cv. Pierre were made in the greenhouse and transplanted in the field in late May of 2020 at the Clifford E. and Melda C. Snyder Research Farm in Pittstown, NJ located at 40° 33’ 33” N and 74° 57’ 31” W. The growing conditions were the same as described by (Patel et al., 2022). The plants were harvested in late August during the full flowering stage. The plants were bulk dried similarly to catnip at 37°C, using the forced air walk-in dryer. Prior to distillation, large stems were removed from the plant material.

The essential oils of catnip cv. CR9 and oregano cv. Pierre were obtained by hydrodistillation as described by Gomes et al. (2020b), with slight modifications. Briefly, 30 g of oregano and 60 g of catnip biomass was added to 1 L of distilled water in a flask that was then heated with electric heating mantles. After 2 h, the essential oils were collected in a Clevenger trap. The essential oils of catnip and oregano were prepared as follows for Gas Chromatography/Mass Spectrometry (GC/MS) analysis: 1 μL of essential oil was added to 1.5 mL of MTBE (methyl-tert-butyl-ether) and dried over anhydrous sodium sulfate (Na_2_SO_4_). The solution was centrifuged at 13,000 rpm for 10 minutes and the supernatant was aliquoted into an amber autosampler vial and used for analysis.

### Essential oil composition *via* GC/MS chemical analysis

The chemical composition of the essential oils of catnip and oregano was determined using a Shimadzu 2010 Plus Gas Chromatograph equipped with a TQ8040 Mass Spectrometer (Shimadzu Scientific, Somerset, NJ). A Restek SH-Rxi-5Sil MS 30 m × 250 μm × 0.25 μm column was used for chromatographic separation. Helium was used as the carrier gas with a column flow rate of 1 mL/min. 1 μL of sample was injected with a split ratio of 25 and the injection temperature was set to 250°C. The column oven temperature was set to 35°C and held at 35°C for 4 min. The temperature was then raised to 250°C at a rate of 20°C/min and held at 250°C for 1.25 min. For the MS parameters, the ion source and interface temperatures were set at 200°C and 250°C respectively. The detector voltage was 0.04 kV and had a threshold of 1000. The solvent cut time was 3.5 min. The fragments were scanned within a range of 45 m/z to 500 m/z. For compound identification, the mass spectra of the compounds were compared to the mass spectra of compounds in the following mass spectral libraries: NIST05.LIB, NIST05s.LIB, W10N14.lib, and W10N14R.lib. To further confirm the identity of the compounds, the mass spectra was compared to Kovats indices generated from a series of n-alkanes (C8-C18) and literature (Adams, 2007). Representative chromatograms and mass spectra of the major compound of catnip and oregano are presented as supplementary material (**Supplementary Figures S1**, **S2**).

### Antibacterial activity assessment

*P. syringae* NJ20-005, *P. cichorii* NJ6-1-2, and *X. perforans* NJ21-014, isolated from various sites in the state of New Jersey, United States were used. The agar well diffusion assay was conducted to test the effects of the catnip and oregano essential oils on *P. syringae*, *P. cichorii* and *X. perforans* by measuring the diameter of the zone of inhibition in mm. Essential oils were serially diluted 2-fold from 40% to 5% in 1 mL of reagent alcohol (90% ethanol, 5% methanol, 5% isopropyl alcohol). Undiluted pure essential oil comprised the 100% concentration treatment, while the negative control consisted of reagent alcohol only. Kanamycin (50 μg/mL) was used as a positive control (Sigma Aldrich, St. Louis, MO). For plate inocula, *P. syringae* and *P. cichorii* were grown on King’s Medium B (KMB) agar (King et al., 1954) and *X. perforans* was grown on yeast-dextrose-calcium carbonate (YDC) agar at 30°C for 48 h. Bacteria were then suspended in sterile distilled water to densities of 1 x 10^8^ CFU/mL, and 100 μl of suspensions of each of the three strains were spread evenly onto their respective media plates. Once the agar was dry of aqueous suspensions, three 6 mm (diameter) wells equidistant from each other were cut into the agar using a cork borer. 60 μL of the treatments were added to each well in a plate. A control well was present in each plate along with two wells containing either the essential oil treatments or the antibiotic treatment. The control well was filled with ethanol for the essential oil treatments and with water for kanamycin. Plates were incubated at room temperature (25°C) for 72 h, and diameters of inhibition zones were measured and reported as means ± standard error (SE). The antibacterial experiments were replicated three times.

### Bed bug petri dish repellency assay

#### Bed bugs

*C. lectularius* Irvington strain, collected from infested apartments in northern New Jersey in 2015, were used for the experiments. The bed bug population was maintained in 4.7 x 4.7 cm plastic containers with harborages made of folded paper. The bed bug colony was maintained at 45 ± 10% RH, and in a photoperiod of 12:12 hours. (L:D) and fed once every three weeks with defibrinated rabbit blood (Hemostat Laboratories, Dixon, CA) using Hemotek membrane-feeding system (Discovery Workshops, Accrington, United Kingdom) but starved for 7-14 days prior to starting the experiments.

#### Essential oil-based repellent preparation

To obtain the oregano dose response curve, oregano essential oil concentrations of 1.25, 2.5, 5 and 10% (w/v) were prepared using reagent alcohol as a vehicle. Based on the results of this study, the dose of 2.5% was chosen for the following experiment as it was the minimum dose at which repellent effect was observed until 24 h. For the next experiment, the repellency of catnip and oregano essential oils, their mixtures and carvacrol were tested at a final concentration of 2.5% (w/v). Previously, the dose of 2.5% of catnip essential oil has also shown excellent repellency up to 24 h in bed bugs (data not shown). The mixtures were included to determine the combined repellent effect of two essential oils that have shown strong repellency against bed bugs previously. Carvacrol, the major constituent of oregano essential oil was also evaluated to determine the repellent effect of the oregano essential oil as whole along with its major compound. The 2.5% (w/v) of catnip and oregano essential oil solutions along with carvacrol (Sigma Aldrich, St. Louis, MO) were prepared in reagent alcohol.

The mixtures of *N. cataria* cv. CR9 (NC) and *O. vulgare* cv. Pierre (OV) essential oils in three different ratios of 25:75 NC : OV, 50:50 NC : OV, 75:25 NC : OV were prepared using reagent alcohol as a vehicle. Mixtures of catnip and oregano essential oil as active ingredients in ratio of 25:75 NC : OV were prepared with 0.625% (w/v) of catnip and 1.875% (w/v) of oregano, mixture with equal parts (50:50 NC : OV) was prepared with 1.25% (w/v) of each essential oil and finally, the mixture of 75:25 NC : OV was prepared with 1.875% of catnip essential oil and 0.625% of oregano essential oil. Concentration-based repellent effects of catnip cv. CR9 have already been established (Reichert et al., 2019; Shi et al., 2021) and as such a dose response curve of catnip essential oil was not evaluated in this study. A 2.5% solution of DEET (N,N-diethyl-meta-toluamide) (Sigma Aldrich, St. Louis, MO), the positive control was also prepared using reagent alcohol which was used as negative control. The solutions were stored in clear, 30 mL plastic spray bottles.

#### Bed bug petri dish assay

100 mm petri dishes were used for the assay (Fisher Scientific, Pittsburgh, PA). Circular 90 mm Whatman No. 1 white filter paper was cut in two equal halves and one half was sprayed with the treatment 4.68 mg/cm^2^, allowed to dry on the bench for approximately 30 min and then glued to the inside surface of a 100 mm petri dish lid with water soluble Elmer’s glue. The two parts of the filter paper were glued to seamlessly fit the petri dish lid and leave no openings for the bed bugs to crawl under. The control side was treated with ethanol in the same way as the treatments. A 20 mm wide harborage tent made from cardstock paper was also treated with the respective treatment and placed on the treated side of the plate. 25 mm tall rings made by removing the bases of deep petri dishes, were coated with talc powder, and placed on the petri dishes with the glued filter paper treatments to prevent the bed bugs from escaping (see **Supplementary Material Figure S4**). Control plates were prepared by spraying one half of the filter paper with ethanol and the other half with water. Ten male adult bed bugs of unknown age were released at the center of the petri dishes for each treatment approximately 30 min after spraying. The petri dishes were placed in larger trays designed to contain the bed bugs and moved to a separate room with 25 ± 2°C, photoperiod of 12:12 (L:D) and 55 ± 5% relative humidity (RH) for observation. The experiment was set up and data was taken during the dark cycle. The number of bed bugs on each side of the petri dish (control vs treatment) was recorded at 1 h and 24 h by visually inspecting the petri dishes. Each treatment was replicated five times. The percentage of repellency was calculated by the proportion of bed bugs repelled relative to the numbers observed in the control petri dishes, using the formula described by (Todd, 2011) for the *in vitro* determination of repellency against bed bugs.

### Statistical analysis

For each experiment, a one-way analysis of variance (ANOVA) was conducted, followed by Tukey’s *post hoc* test. For quantitative treatments, regression analyses were performed, and the models were chosen based on the highest coefficient of determination (**Supplementary Figures S3**, **S5**). GraphPad Prism software version 9.4.1 was used to perform all statistical analyses.

## Results

### Essential oil composition

In the essential oil of catnip cv. CR9, nine compounds were identified and in total, the identified peaks represented 94.46% of the total peak area of the essential oil profile. The major constituent of the essential oil of *N. cataria* cv. CR9 was (*Z,E*)-nepetalactone (84.3%), followed by *β*-caryophyllene (6.73%) and *β*-pinene (2.04%). Some other important compounds also found in the catnip essential oil but in amounts of less than 0.5% are *α*-humulene and caryophyllene oxide. Oregano essential oil showed a more complex profile, with fifteen peaks identified, representing 97.66% of the total peak area. The major constituent of the essential oil of *Origanum vulgare* cv. Pierre was carvacrol (61.9%), followed by p-cymene (25.2%) and γ-terpinene (2.24%). Additionally, some other compounds such as thujene, α -pinene, camphene, *β-*myrcene, α-terpinene, *β-*caryophyllene, α-humulene, *β*-bisabolene and caryophyllene oxide were also present in the oil. In both catnip and oregano essential oils, monoterpenes are the major constituents of the oil, followed by sesquiterpenes (**Table 1**).

**Table 1:** Relative percent peak areas of the chemical constituents of the essential oil of *Origanum vulgare* cv. Pierre and *Nepeta cataria* cv. CR9 obtained by Gas Chromatography/Mass Spectrometry.

ID #	RI	R_T_	Compound	% Peak Area ± SD	
				Catnip cv. CR9	Oregano cv. Pierre
**1**	862	6.761	Unidentified 1	1.07 ± 0.1	1.03 ± 0.02
**2**	926	7.641	Thujene	–	T
**3**	935	7.739	*α*-pinene	T	0.69 ± 0.01
**4**	952	7.944	Camphene	–	T
**5**	973	8.196	Unidentified 2	T	–
**6**	977	8.235	1-Octen-3ol	–	0.55 ± 0.04
**7**	979	8.263	*β*-pinene	2.04 ± 0.04	–
**8**	983	8.300	Unidentified 3	T	T
**9**	986	8.345	*β*-myrcene	–	0.97 ± 0.03
**10**	1017	8.662	*α-*terpinene	–	0.62 ± 0.03
**11**	1024	8.736	p-cymene	–	25.2 ± 0.35
**12**	1030	8.789	Unidentified 4	T	–
**13**	1032	8.810	*Trans β*-ocimene	T	0.96 ± 0.04
**14**	1059	9.063	γ-terpinene	–	2.24 ± 0.03
**15**	1095	9.408	Unidentified 5	–	T
**16**	1178	10.111	Endo-borneol	–	0.80 ± 0.05
**17**	1185	10.162	Unidentified 6	T	0.54 ± 0.03
**18**	1294	11.008	Carvacrol	–	61.9 ± 0.25
**19**	1371	11.649	(*Z,E*)-nepetalactone	84.3 ± 0.22	–
**20**	1407	11.806	(*E,Z*)-nepetalactone	T	–
**21**	1434	11.985	*β*-caryophyllene	6.73 ± 0.10	1.01 ± 0.02
**22**	1445	12.056	*β*-farnesene	0.10 ± 0.01	–
**23**	1470	12.222	*α*-humulene	T	T
**24**	1507	12.462	*β*-bisabolene	–	0.99 ± 0.02
**25**	1514	12.503	Unidentified 7	3.67 ± 0.06	T
**26**	1598	13.042	Caryophyllene oxide	T	1.07 ± 0.05
			**Total Identified Peaks**	94.46	97.66
			**Total Unidentified Peaks**	5.53	2.33

The relative peak percentage areas of the compounds are presented as the mean of three replicates **±** Standard Deviation. Compounds that were not detected are indicated by [**-**]. RI, Kovats retention indices; R_T_, Retention Time; T, Trace amounts (less than 0.5%).

### Bacterial growth inhibition assay

**Figure 1:**
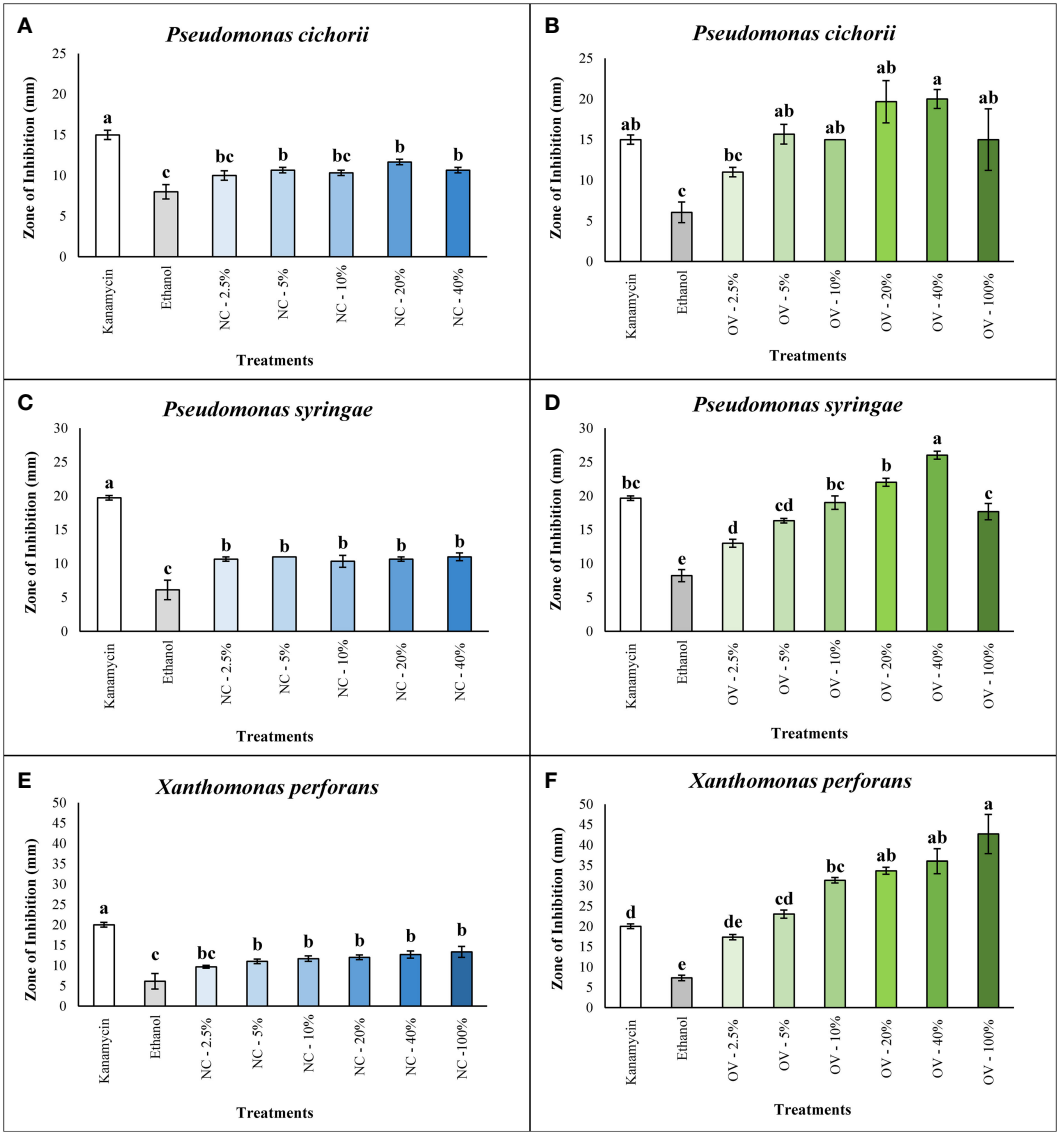
Antibacterial activity of *Nepeta cataria* cv. CR9 (NC) and *Origanum vulgare* cv. Pierre (OV)’s essential oils at various concentrations of 2.5%, 5%, 10%, 20%, 40% and 100% (v/v) measured by the zone of inhibition (mm) against *Pseudomonas cichorii*, *Pseudomonas syringae*, and *Xanthomonas perforans* in the agar well diffusion assay. Antibacterial activity of NC against *P. cichorii* **(A)**. Antibacterial activity of NC against *P. syringae* **(C)**. Antibacterial activity of NC against *X. perforans* **(E)**. Antibacterial activity of OV against *P. cichorii* **(B)**. Antibacterial activity of OV against *P. syringae* **(D)**. Antibacterial activity of OV against *X. perforans* **(F)**. Means followed by the same letters in the columns do not differ statistically according to the Tukey test at 5% probability level. The error bars represent the standard error of mean.

Essential oils of catnip and oregano were tested for *in vitro* growth inhibition against the three different bacterial plant pathogens. Oils were evaluated at six different concentrations using an agar well diffusion assay. Growth inhibition was not observed for *P. cichorii and P. syringae* treated with pure (100%) catnip essential oil, and results were included in the regression analyses in supplementary material (**Figure S3**). While growth inhibition was not observed using the pure essential oil, lower concentrations of the oils showed significant activity that resulted in inhibition zones ranging from 10 ± 0.6 mm to 11.7 ± 0.3 mm for *P. cichorii*, exposed to catnip essential oil at concentrations of 5%, 20% and 40%. While growth inhibition was statistically superior to the negative control (p ≤ 0.05), they were inferior to kanamycin at 50 µg/mL (**Figure 1A**). Regression analysis for the concentrations of catnip oil against *P. cichorii* presented a quadratic behavior, evidenced by the slight decrease in inhibition observed at 40% and marked decrease with the pure essential oil (100%) (**Supplementary Figure S3A**).

The responses of *P. syringae* to different concentrations of catnip essential oil were similar to *P. cichorii*. All the doses ranging from 2.5% to 40% did not differ significantly from each other but they all performed better than the ethanol negative control (p ≤ 0.05) yet not as well as the positive control antibiotic kanamycin at 50 µg/mL (**Figure 1C**). Catnip essential oil concentrations presented intermediate inhibitions zones, ranging from 10.3 ± 0.9 mm to 11.0 ± 0.6 mm (**Figure 1C**). Regression analysis of different catnip oil concentrations for *P. syringae* is presented in supplementary material (**Figure S3C**) and, similarly to *P. cichorii*, shows a quadratic behavior, mainly characterized by the steep decline in the antimicrobial activity of the pure essential oil.

Values of zones of inhibition for *X. perforans* were larger overall compared to those for the two *Pseudomonas* species tested in this study. Inhibition zones occurring with doses of catnip essential oil ranged from 9.7 ± 0.3 mm to 13.3 ± 1.3 mm but did not reach levels observed with the antibiotic kanamycin, which showed the largest zone of inhibition (20 ± 0.6 mm). However, the exception of the 2.5% dose, all other doses from 5% to 100% were significantly greater that the negative control of ethanol alone (6.1 ± 1.9 mm) (**Figure 1E**). In the regression analysis for *X. perforans*, the reduced inhibition with the pure essential oil was not observed, fitting a logarithmic model in the regression analysis (**Supplementary Figure S3E**). Overall, catnip essential oil at specific concentrations showed significant antibacterial activity against the three bacterial species in this study (**Figures 1A, C, E**).

The essential oil of oregano cv. Pierre showed higher antibacterial activity against all three bacterial plant pathogens compared to essential oil of catnip cv. CR9. For *P. cichorii*, average zones of inhibition reached 20 ± 1.2 mm at concentrations of 40%, which was statistically superior to the oil at 2.5% concentration and ethanol control (6.1 ± 1.3 mm for reference) (**Figure 1B**). Inhibition zones resulting from essential oil concentrations at 5% and greater were statistically equivalent to kanamycin at 50 µg/mL, although average zone size resulting for 20% and 40% concentrations were higher than that of the antibiotic (**Figure 1B**).

Compared with *P. cichorii*, essential oil of oregano showed higher antibacterial activity towards both *P. syringae* and *X. perforans*. For *P. syringae*, the essential oil at a concentration of 40% had the highest zone of inhibition (26 ± 0.6 mm) and was significantly greater to all other treatments including kanamycin at 50 µg/mL (**Figure 1D**). Oregano essential oil concentrations at 5% or greater demonstrated antibacterial activity compared to the ethanol control (8.2 ± 0.9 mm). More importantly, concentrations at 5, 10, 20 and 100% were statistically equivalent to kanamycin (**Figure 1D**). Regression analyses for both *P. cichorii* and *P. syringae* showed a clear concentration-dependent effect, with a quadratic behavior characterized by a peak bioactivity between 20% and 40% and the reduction of the average zone of inhibition with the use of pure oil (100%), although not as steep as observed for catnip oil (**Supplementary Figure S3B, S3D**).

Oregano essential oil showed the highest antibacterial activity towards *X. perforans*. All concentrations of oregano essential oil at 5% and higher displayed zones of inhibition significantly greater than the ethanol alone control (**Figure 1F**). The level of inhibition was equivalent to kanamycin (50 µg/mL) at 5% concentration and was statistically greater for concentrations of 10% and higher (**Figure 1F**). Interestingly, the pure essential oil of oregano showed the greatest average size of zone of inhibition of *X. perforans*, although it did not differ statistically from 20 and 40% concentrations.

Both catnip and oregano essential oils exhibited a significantly higher antibacterial activity compared to the ethanol control in all three species at the dose of 5% or above (**Figure 1**). Catnip and oregano essential oils had superior antibacterial activity to ethanol even at the dose of 2.5% against *P. syringae* (**Figures 1C, D**). Catnip essential oil displayed a significant antibacterial activity, although lower than that of the oregano essential oil which had an equal or significantly higher antibacterial activity at doses of 2.5% or above (**Figure 1**).

### Bed bug repellency

**Figure 2:**
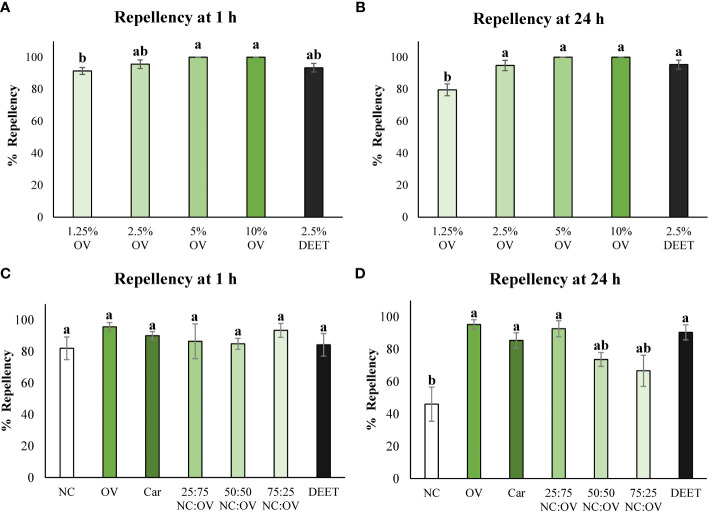
Repellency of *Origanum vulgare* cv. Pierre (OV) essential oil at various concentrations (1.25, 2.5, 5 and 10%) against *Cimex lectularius* (common bed bug) at 1 h **(A)** and 24 h **(B)**. Repellency of *Nepeta cataria* cv. CR9 (NC) and *Origanum vulgare* cv. Pierre (OV) essential oils along with their mixtures at various ratios (NC:OV) and Carvacrol (Car), the major compound found in oregano essential oil at concentration of 2.5% against *Cimex lectularius* (common bed bug) at 1 h **(C)** and 24 h **(D)** using the petri dish assay. Columns with the same letters do not significantly differ from each other according to Tukey’s test (p>0.05). Error bars represent the standard error of mean.

For the first experiment, the dose response of different concentrations of oregano essential oil was evaluated to find the minimum concentration needed to exhibit repellency against bed bugs. After 1 hour in the petri dish, concentrations of 5% and 10% doses of oregano essential oil showed 100% repellency, 2.5% essential oil showed 96 ± 3% repellency and 1.25% and DEET exhibited 91 ± 2% and 93 ± 3% repellency respectively. There were no statistically significant differences amongst the concentrations of 2.5%, 5%, 10% and DEET treatments. 1.25% concentration was statistically inferior from higher oregano essential oil concentrations, although still presenting remarkably high repellency (**Figure 2A**).

After 24 hours of exposure, similar results were observed to those verified at 1 hour, with concentrations of oregano oil of 2.5% and above and DEET showing almost complete repellency and not differing statistically from each other. The concentration of 1.25% of essential oil still remained showing high repellency, although not as repellent as higher the concentrations and DEET (**Figure 2B**). The regression analyses for repellency show that the data fits a logarithmic curve at both 1 hour and 24 hour time points as it is characterized by an increase in repellency up until the dose of 2.5% and then the repellency stabilizes for the last two doses of 5 and 10% (**Supplementary Figure S5**).

The following experiment evaluated the effects of catnip and oregano essential oils, their mixtures and carvacrol at the concentration of 2.5% as this dose was determined to the minimum dose necessary in the previous oregano dose response experiment to see a repellent effect in bed bugs. At 1 hour, all the treatments were statistically equivalent to each other (**Figure 2C**). At 24 hours the catnip essential oil still possessed repellency however, it was inferior to that of the oregano essential oil, the oregano and catnip mixtures as well as carvacrol and DEET which did not differ statistically from each other (**Figure 2D**).

## Discussion

The aim of this study was to determine the antibacterial and insect repellent activities of catnip cv. CR9 and oregano cv. Pierre. The major component of the essential oil from novel catnip cultivar CR9 is *(Z,E)-nepetalactone*, a bicyclic oxygenated monoterpene that has shown to have remarkable insect repellent activity against malaria vectoring mosquitos, ticks, chicken mites, houseflies, and stable flies, among others (Gomes et al., 2020a). The chemical profile of catnip essential oil is consistent with the profiles reported in literature with *(Z,E)-nepetalactone* as the major constituent, followed by minor components such as *β*-caryophyllene and caryophyllene oxide, whose amounts vary (Reichert et al., 2016; Reichert et al., 2019). Nepetalactone and its various isomers have been reported to be the major constituent of *N. cataria* essential oil as well as the oil of other species of catnip in the *Nepeta* genus (Zenasni et al., 2008; Asgarpanah et al., 2014; Gomes et al., 2020a). Catnip genotypes possess distinct chemical profiles, with some catnip genotypes such as lemon catnip, lack the major compound nepetalactone (Gomes et al., 2020b). Therefore, having a genotype that produces high yield of nepetalactone is key for steady supply of effective products. Thus, catnip cv. CR9, a nepetalactone rich chemotype containing a single isomer of *(Z,E)-*nepetalactone as its major compound along with other desirable agronomic traits (Reichert et al., 2016), provides reliable genetic materials suitable for industrial applications.

Carvacrol was the most abundant compound in the essential oil of new oregano cv. Pierre, followed by p-cymene, a profile consistent with previously reported analyses (Reichert et al., 2021). Carvacrol is a commercially valuable molecule possessing antimicrobial, antioxidant and anti-inflammatory properties and has been approved by the United States (US) Food and Drug Administration (FDA) for use in food and as a chemical flavoring by the Council of Europe (Zotti et al., 2013). The high carvacrol yielding oregano cultivar Pierre, developed for the Mid-Atlantic region in Northern America (Reichert et al., 2021) can provide a reliable supply of essential oil with a consistent chemical profile to support the growing flavors and fragrance market which is projected to reach $5.4 billion in sales by 2025 in the United States (Statista, 2022). *P. cichorii* and *P. syringae* are bacterial plant pathogens that negatively impact economically valuable crops (Lamichhane et al., 2015). Both the catnip and oregano essential oils showed inhibitory activity against these two *Pseudomonas* species. The catnip essential oil showed significant antibacterial activity, however not as strong as the oregano oil, which was even superior to the antibiotic kanamycin in concentrations above 10%. Thus, its potential to serve as an antibacterial agent can be further evaluated against other plant pathogens of interest using the same or different methods. Essential oil and ethanolic extracts of novel catnip cv. CR9 have previously showed repellent activities against mosquitoes, ticks, and bed bugs (Reichert et al., 2019; Shi et al., 2021; González et al., 2022), but it is reported in this study for the first time that the essential oil from this genotype also displays antibacterial activity.

The antibacterial activity of essential oils from other *N. cataria* genotypes has previously been tested against food borne pathogens, respiratory tract pathogens and skin commensals, such as *S. aureus, E. coli, P. aeruginosa, Klebsiella pneumoniae*, and *Staphylococcus epidermidis* (Suschke et al., 2006; Suschke et al., 2007; Zenasni et al., 2008; Adiguzel et al., 2009; Zomorodian et al., 2012; Vukovic et al., 2016; Ashrafi et al., 2019). For *Pseudomonas* species, while the antibacterial effects of extracts and oils of *N. cataria* against *P. syringae* have been tested previously (Adiguzel et al., 2009), the inhibitory effects of catnip essential oil against *P. cichorii* are presented here for the first time. Essential oil of oregano cv. Pierre showed overall a stronger antibacterial activity than catnip cv. CR9 and, in higher concentrations, was even superior to the antibiotic kanamycin. Oregano essential oil has a well-established history of use as an antibacterial agent for applications in the food industry (Rodriguez-Garcia et al., 2016), and its activity against *P. syringae* and its various strains has been well documented (Vasinauskiene et al., 2006; Carezzano et al., 2017). However, similarly to the information previously discussed for catnip, to the best of our knowledge, there are no reports of the use of oregano essential oil against *P. cichorii*.

An interesting trend observed for both catnip and oregano was a reduction, with varying degrees of intensity, in the zones of inhibition of wells filled with the pure (100%) essential oils, in comparison to diluted essential oils, mainly at 20 and 40% concentrations, for both *P. syringae* and *P. cichorii*, but not for *X. perforans*, when the pure essential oils performed similarly or better than when diluted. One possible explanation is that the use of pure essential oil hinders its diffusion through the media due to having higher viscosity compared to the concentrations diluted with ethanol. More viscous essential oils have been reported to exhibit little antibacterial activity compared to less viscous oils in the agar well diffusion assay (Hood et al., 2003). The reason why *X. perforans* appears to be less affected by such phenomena is possibly related to the differences in media composition. The *Pseudomonas* species used in this study were cultured in King’s Medium B (KMB), while *X. perforans* was cultured in Yeast-Dextrose-Calcium Carbonate (YDC) medium. One of the main differences among these media that can affect the diffusion of viscous liquids is that KMB contains glycerol, which is a viscous liquid in itself, its presence in the medium may impede the diffusion of the essential oils. Additionally, previous studies have also reported different susceptibility among bacteria species to essential oil properties, some being inhibited more by vapor absorption (not directly dependent on media diffusion) and some being more susceptible to direct contact through the media (Inouye et al., 2001).

Despite the differences observed in the treatments with *P. cichorii and P. syringae*, when using the pure essential oil, *X. perforans* was also inhibited by catnip and, especially by oregano essential oils. Previously carvacrol, a major component of essential oil of oregano and in particular the major component of oregano cv. Pierre’s essential oil has been tested against copper resistant *X. perforans* in *in vitro*, greenhouse and field settings (Qiao et al., 2020). Carvacrol has shown to increase the sensitivity of the resistant *X. perforans* to copper-based treatments, promoted germination of tomato seeds, increased seedling vigor, reduced severity of bacterial spot and enhanced efficacy of copper against the resistant *X. perforans* strain (Qiao et al., 2020). As for catnip, previous studies of antimicrobial activity have been reported in other *Xanthomonas* species (Kokoskova and Pavela, 2006; Adiguzel et al., 2009), but this is the first report of inhibitory effects of catnip essential oil against *X. perforans.* The strong inhibitory effect displayed by the oregano essential oil suggests that it can be an excellent candidate for further studies to evaluate its potential in other *in vitro* tests as well as in agronomic evaluations of disease incidence and severity.

Different essential oils have been assessed for their antimicrobial activities, with mixed results (Ahluwalia et al., 2014; Mandal et al., 2021; Manjesh et al., 2022). These natural products are reported to have multiple modes of action that involve damage to the cell wall and membrane, changes to the fatty acid composition, leakage of ions and intracellular metabolites, disruption in glucose uptake and metabolic activity among others (Nazzaro et al., 2013; Angane et al., 2022; Chang et al., 2022). One of the many reported modes of action of oregano essential and oil and carvacrol, is their ability to permeabilize bacterial cell membranes (Hyldgaard et al., 2012). The efficacy of essential oils can be enhanced due to the synergy between their various components (Isman et al., 2011), making them ideal candidates for developing new antibacterial agents and crop protectants to combat resistant pathogens and pests.

In a study by Gavaric et al. (2015), both combinations of oregano and thyme essential oil as well as those of carvacrol and thymol, their major components, had an additive effect when tested against *S. aureus*, *B. cereus*, *E. coli* and *Salmonella infantis*. Carvacrol and thymol have also exhibited additive effects against *S. aureus* and *P. aeruginosa* (Lambert et al., 2001). A mixture of (*Z,E*)-nepetalactone and (*E,Z*)-nepetalactone has exhibited synergistic effects and antibacterial activity against five different *Neisseria* spp. (Ghosh et al., 2021). Literature regarding the potential synergistic, additive, or antagonistic interactions between the major and minor components of *N. cataria* essential oil against bacterial pathogens is scarce. Thus, additional studies need to be conducted to understand the type of interactions between the components and their effect on the overall activity of the oil.

In addition to carvacrol and nepetalactone, other compounds in the essential oils of catnip and oregano can influence their antimicrobial activity, either synergistically, additively, or antagonistically. Synergism between the major component of oregano, carvacrol and other components such as p-cymene, a weak antibacterial compound that is also a precursor of carvacrol, has been observed against *B. cereus in vitro* and in foods such as rice (Burt, 2004). The combination of carvacrol and cymene has shown to inhibit growth of *V. cholerae* in food, with cymene reportedly increasing the inhibitory activity of carvacrol (Rattanachaikunsopon and Phumkhachorn, 2010). The effectiveness of the combination of carvacrol and cymene may be due to the ability of cymene to expand the membrane, allowing an increase in the amount of carvacrol transported into the cell (Burt, 2004). In addition to p-cymene, γ-terpinene has also been reported to show varying levels of activity against several bacterial pathogens (Aligiannis et al., 2001; Cristani et al., 2007). Contrastingly, antagonistic effects of the combinations of carvacrol/thymol, carvacrol/eugenol and carvacrol/myrcene have also been reported in *S. aureus*, *B. cereus* and *E. coli*, with myrcene alone exhibiting no inhibitory activity against these pathogens (Gallucci et al., 2009).

Low molecular weight, hydrophobic terpenoids with weak antimicrobial activity of their own are reported to contribute to the overall antimicrobial activity of the essential oils due to their ability to disrupt the cell wall and enable the more active components to enter the cell (Álvarez-Martínez et al., 2020). The essential oil of oregano cv. Pierre has carvacrol as its major compound, followed by the terpenes p-cymene and γ-terpinene. It is possible that some of these compounds and other minor ones contribute to the observed antibacterial activity of the oregano oil. Future studies with the whole essential oil of oregano cv. Pierre as well as its individual constituents may help in further elucidating the potential synergism/antagonism between the components of the oil and their impact on the bioactivity of the oil. In contrast to the essential oils, the antibiotic kanamycin, belonging to the aminoglycosides class of compounds, leads to inhibition of protein synthesis by misreading the nucleotides and terminating the translation of mRNA (Dowling et al., 2017; Kapoor et al., 2017).

Essential oils can also affect the physiology and behavior of insects, with mono and sesquiterpenoid components of essential oils exhibiting sublethal effects on arthropods such as oviposition deterrence and repellency (Chang et al., 2022). In addition to demonstrating promising antibacterial effects, catnip cv. CR9 and oregano cv. Pierre essential oils also showed remarkable repellency against the common bed bug (*C. lectularius*). Catnip has been previously shown to repel bed bugs in a concentration-dependent manner (Shi et al., 2021), so in the present study only the novel oregano essential oil was studied to find the optimal concentration for repellency. Concentrations as low as 2.5% were found to be enough to cause complete repellency in petri dish assays, with results comparable to equivalent doses of DEET, insect repellent’s gold standard. Two isomers of nepetalactone [(4aS,7S,7aR) and (4aS,7S,7aS)] showed lower repellency against *Anopheles gambiae* compared to the catnip oil, indicating potential synergism between them, and when combined with (E)-(1R,9S)-caryophyllene, the resulting mixture was reported to have the same repellent activity as the original catnip oil (Birkett et al., 2011).

Previously, the essential oil from a different oregano genotype, mainly composed of terpineol and α-terpinene, has been tested against bed bugs in the petri dish repellency assay, repelling only 10% of the insects after 24 hours with a concentration of 2.5% (Sharififard et al., 2018). In the present study, the same essential oil concentration of oregano cv. Pierre showed 100% repellency. Such a contrasting result can be explained by the unique chemical composition of oregano cv. Pierre, which produces high levels of carvacrol and p-cymene, compounds widely regarded as strong repellents of different insects (Evergetis et al., 2018; Feng et al., 2021). The concentration of 1.25% of oregano cv. Pierre still showed high repellency after 24 hours of exposure in the petri dishes, although inferior to DEET and higher concentrations of the essential oil. In addition to repellency, oregano essential oil has been reported to display toxicity to bed bugs when applied topically in a study by Zha et al. (2018), however the chemical composition of the oregano oil used in the study is unknown. A study by Gaire et al. (2020a) demonstrated significant insecticidal activity of oregano essential oil against a deltamethrin resistant bed bug strain Knoxville. In another study, topically applied binary mixture of oregano essential oil and deltamethrin significantly increased mortality in resistant bed bugs (Gaire et al., 2021). The use of oregano essential oil as a repellent has been reported against other pests of importance to public health such as vectors of infectious diseases (mosquitoes and ticks), head lice and brown-banded cockroach (Alizadeh et al., 2021), however this is the first time that a carvacrol-rich oregano essential oil is reported to repel bed bugs.

After defining that the concentration of 2.5% was equivalent to higher concentrations of oregano essential oil, a subsequent study was conducted to assess the effectiveness of catnip and oregano mixtures along with carvacrol, the major component of oregano cv. Pierre’s essential oil as bed bug repellents at that concentration. Catnip, oregano, and their mixtures showed strong repellency against bed bugs after 1 hour of exposure, being equivalent to DEET. After 24 hours in the petri dishes, catnip essential at the 2.5% concentration shows a reduction in its bioactivity, being inferior to DEET and oregano essential oil. However, the different mixtures containing oregano remain equivalent to DEET at 24 hours, despite slight reductions in the average repellency, evidencing that combining catnip and oregano essential oils may help to increase the duration of repellency of products containing essential oil of catnip cv. CR9.

In this study, carvacrol showed repellency similar to that observed in the essential oil of oregano, demonstrating that it is the main contributor to the activity of the essential oil of oregano against *C. lectularius.* Additionally, carvacrol has demonstrated considerable topical and fumigant toxicity as well as significant neuroinhibitory effects in susceptible bed bugs (Gaire et al., 2019) and enhanced deltamethrin toxicity in resistant bed bugs (Gaire et al., 2021). Furthermore, carvacrol has shown to act synergistically when combined with other monoterpenoids such as thymol and eugenol in topical bioassays in bed bugs (Gaire et al., 2020b). Carvacrol exhibited acute topical toxicity against *Musa domestica* adults and showed significant synergy in the topical application assay when combined with p-cymene (Pavela, 2008). In another study by Pavela (2015), acute toxicity against the larvae of *Culex quinquefasciatus* was observed under the synergistic combinations of carvacrol and terpineol, carvacrol and carvone, and carvacrol and 4-allylanisole. In terms of repellency, carvacrol has been previously reported to have a repellent effect against bed bugs for up to 24 hours in the petri dish repellency assay (González-Morales et al., 2021).

Catnip cv. CR9’s essential oil has shown to be an effective repellent against ticks and mosquitoes, and it has also been tested previously in bed bugs (Shi et al., 2021). In addition, catnip essential oil and its main constituent nepetalactone have shown to repel other insects such as stable flies, house flies, ticks, chicken mites and many species of mosquitoes that vector diseases such as malaria (Gomes et al., 2020a). Catnip cv. CR9 offers a solution to challenges faced previously regarding catnip cultivation and essential oil production as it is a nepetalactone rich cultivar with high biomass yield and upright growth habit which is ideal for mechanical harvesting (Reichert et al., 2016; Gomes et al., 2020a). Catnip can be grown locally in rural communities as well to diversify crops and generate extra income, in addition to providing a source of insect repellents in areas where synthetic repellents may be hard to purchase (Gomes et al., 2020a). The oregano cv. Pierre can offer a high-quality source of oregano essential oil with a stable chemical profile for cultivation in the Mid-Atlantic Region of the United States (Reichert et al., 2021). With numerous applications in the food, beverage, personal care, cosmetics, and pharmaceutical industries, growing oregano can be profitable due to low cost of cultivation and increasing market demand for oregano oil (Dordas, 2009; Nazar et al., 2022). The strong repellency of both catnip and oregano essential oils against bed bugs makes them excellent candidates for plant-based insect repellents that can provide a more sustainable way to manage pests of human and agricultural importance.

## Conclusion

Novel catnip cv. CR9 exhibits an essential oil dominated by (*Z,E*)-nepetalactone and minor compounds such as *β*-pinene, *β*-caryophyllene, *α*-humulene and caryophyllene oxide. Oregano cv. Pierre presents carvacrol as its major component, followed by p-cymene and has minor compounds such as α-pinene, *β-*myrcene, γ-terpinene, *β-*caryophyllene, α-humulene, *β*-bisabolene and caryophyllene oxide. Both essential oils from catnip and oregano demonstrated antibacterial effects against *P. cichorii*, *P. syringae*, and *X. perforans*. Catnip essential oil concentrations of at least 5% are needed to inhibit *P. cichorii* and *X. perforans*, while 2.5% is enough to inhibit *P. syringae*. Oregano essential oil had a stronger inhibitory effect than catnip essential oil, with concentrations of at least 10% being equivalent or superior to the antibiotic kanamycin.

The essential oil of Oregano cv. Pierre at the concentration of 2.5% repels bed bugs as effectively as DEET after 24 hours of exposure in petri dish repellency assays. Mixture containing 25% catnip essential oil and 75% oregano essential oil works more effectively than catnip alone and is as effective as oregano essential oil alone and DEET. Carvacrol shows an activity similar to the essential oil of oregano, evidencing that it is a major contributor to the repellent activity of oregano essential oil against bed bugs. Essential oils from the novel cultivars of catnip and oregano can aid in the development of products for managing pathogens and pests in agricultural and public health sectors. Future studies can evaluate the effects of these natural products in *ex vitro* and field settings as well as consider their effects on non-target organisms such as beneficial microbes, insects, and humans.

## Data availability statement

The raw data supporting the conclusions of this article will be made available by the authors, without undue reservation.

## Author contributions

HKP, ENG, NP, CW, and JES: Conceptualization. HKP, ENG, NP, DYK, and CW: Methodology. HKP and ENG: Data analysis. HKP and ENG: Original draft preparation. NP, DYK, CW, QW, and JES: Review and editing. QW and JES: Supervision and funding acquisition. All authors contributed to the article and approved the submitted version.
