# Supplementary material for: Volatile metabolites from new cultivars of catnip and oregano as potential antibacterial and insect repellent agents
Source: Front Plant Sci. 2023 Feb 23;14:1124305. doi: 10.3389/fpls.2023.1124305 (PMC9995836; doi:10.3389/fpls.2023.1124305)
Supplement: Supplementary file 1 [file DataSheet_1.pdf]

Supplementary Material

Volatile metabolites from new cultivars of catnip and oregano as potential antibacterial and insect repellent agents

Harna K. Patel[1,2], Erik Nunes Gomes[1,2,3], Qingli Wu[1,2,4], Nrupali Patel[2], Donald Y. Kobayashi[2], Changlu Wang[5], James E. Simon[1,2,4,6*]

[1]New Use Agriculture and Natural Plant Products Program, Department of Plant Biology, Rutgers University, New Brunswick, NJ, USA

[2]Department of Plant Biology, Rutgers University, New Brunswick, NJ, USA

[3]Federal Agency for Support and Evaluation of Graduate Education (CAPES), Ministry of Education of Brazil, Brasilia 70.040-020, DF, Brazil

[4]Department of Medicinal Chemistry, Ernest Mario School of Pharmacy, Rutgers University, Piscataway, NJ, USA.

[5]Department of Entomology, Rutgers University, New Brunswick, NJ, USA

[6]Center for Agricultural Food Ecosystems, Institute of Food, Nutrition & Health, Rutgers University, New Brunswick, NJ, USA

*** Correspondence:**
James E. Simon
jimsimon@rutgers.edu

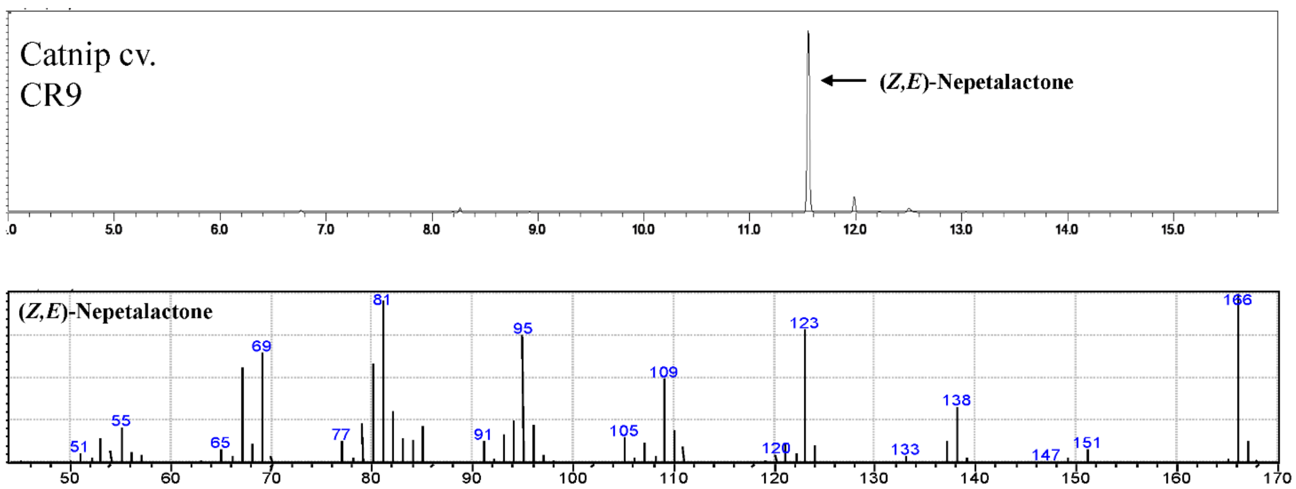

Figure S1. Representative GC chromatogram of the essential oil of catnip cv. CR9 followed by the mass spectra of the major compound of the essential oil, *(Z,E)*-nepetalactone.

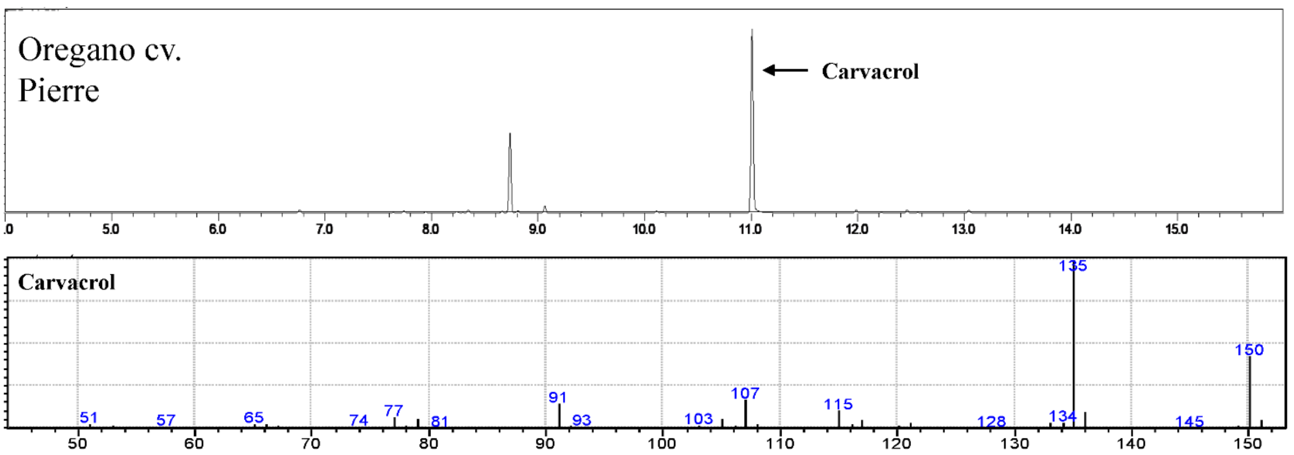

Figure S2. Representative GC chromatogram of the essential oil of oregano cv. Pierre followed by the mass spectra of the major compound of the essential oil, carvacrol.

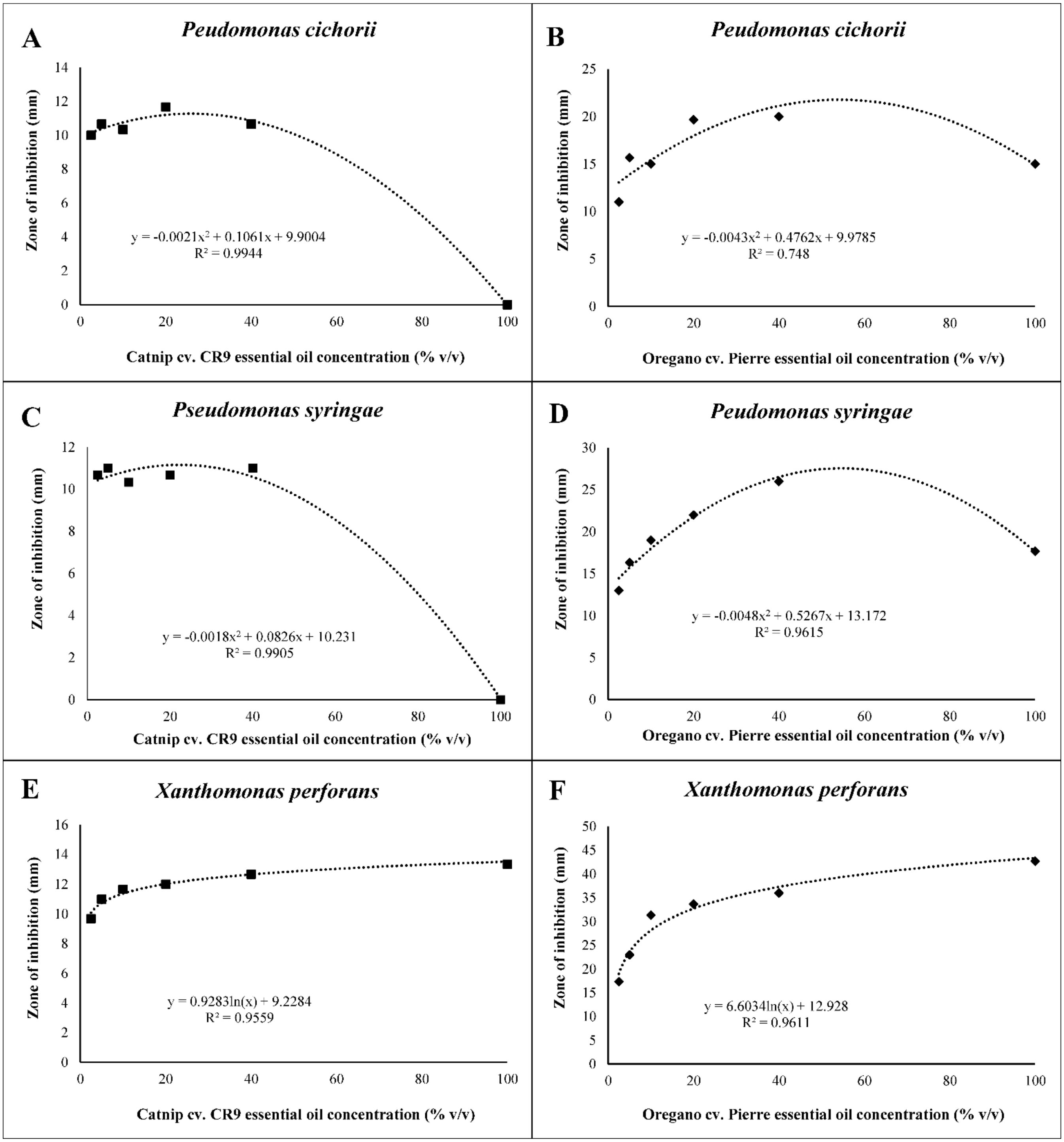

Figure S3. Regression analysis of the antibacterial activity of catnip cv. CR9 and oregano cv. Pierre against *Pseudomonas cichorii*, *Pseudomonas syringae* and *Xanthomonas perforans*. The antibacterial activity was measured as the zone of inhibition (mm). The dose response curve of catnip cv. CR9 and oregano cv. Pierre essential oils was created using concentrations of 2.5%, 5%, 10%, 20%, 40% (v/v) and 100% (pure essential oil).

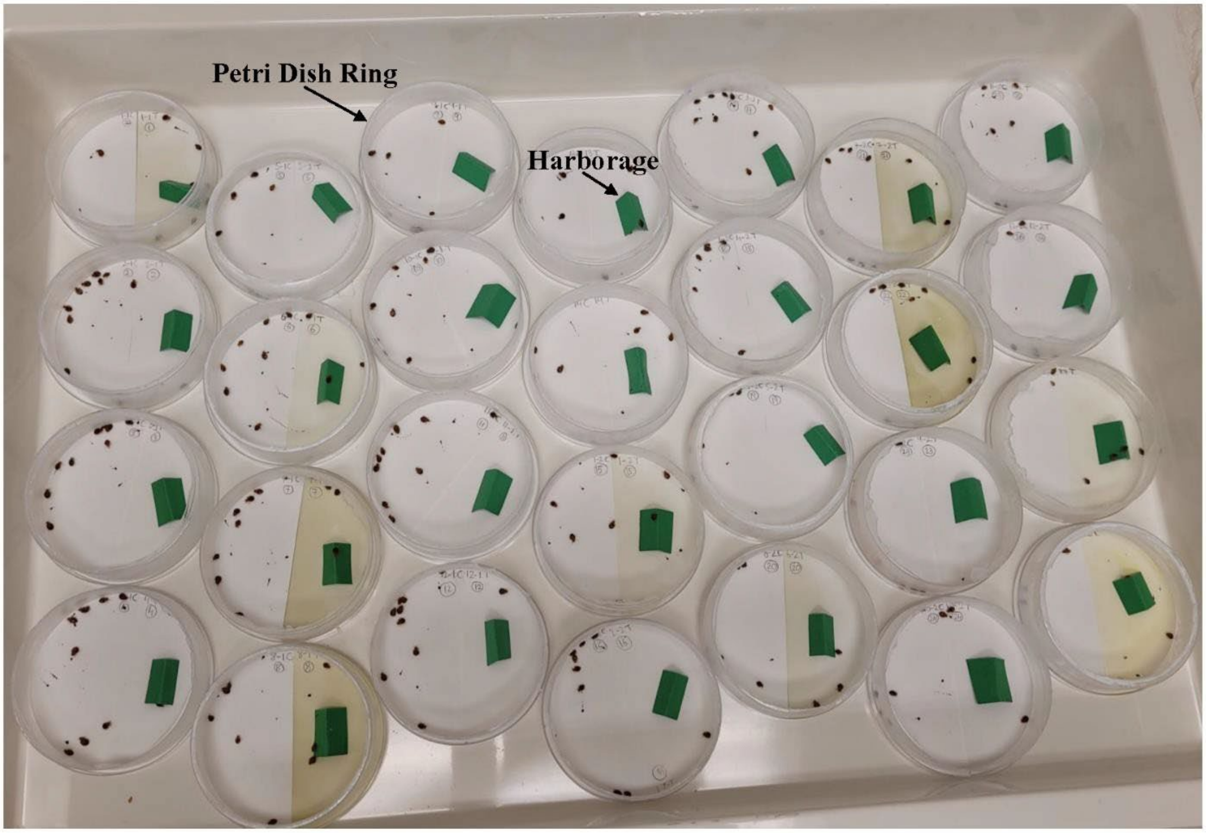

Figure S4. Set up of the bed bug petri dish repellency assay. Petri dishes with various treatments are placed in a larger tray. Each petri dish consists of a control and treatment side with a 2 cm harborage placed on the treatment side. A 25 mm plastic ring coated with talcum powder is placed in the dish to prevent bed bugs from escaping the dish.

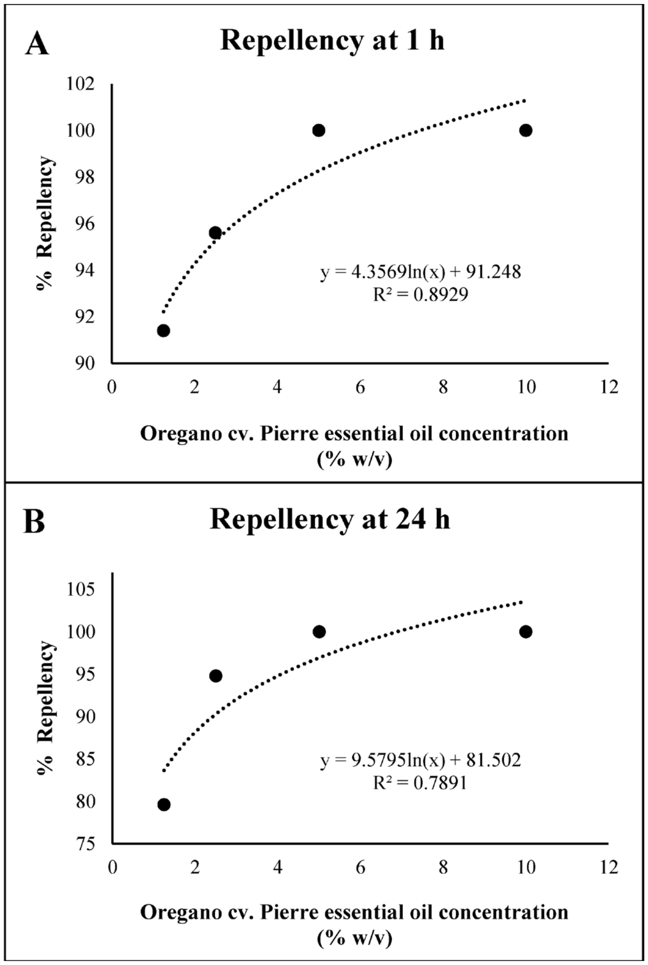

Figure S5. Regression analysis of various concentrations of the essential oil of oregano cv. Pierre at 1 h and 24 h. The repellent effect was measured as % repellency of the dose response curve of the essential oil of oregano cv. Pierre at 1 and 24 h against the common bed bug (*Cimex lectularius*).
